# Comparison of Endophthalmitis Rates Between Prefilled Syringes and Standard Vials in Aflibercept Intravitreal Injections: A Retrospective Study in Japan

**DOI:** 10.3390/jcm14072491

**Published:** 2025-04-06

**Authors:** Masakazu Morioka, Yoshihiro Takamura, Shigeo Yoshida, Junya Mori, Tomoko Sawada, Hisashi Matsubara, Sentaro Kusuhara, Tomoya Murakami, Aki Kato, Hitoshi Tabuchi, Daisuke Nagasato, Tetsuo Ueda, Masahiko Shimura, Takao Hirano, Tatsuya Jujo, Yoshinori Mitamura, Masashi Nishigaki, Kozo Harimoto, Mariko Sasaki, Masaru Inatani

**Affiliations:** 1 J-CREST (Japan Clinical REtina STudy Group), Kagoshima 890-8544, Japan; mmorioka@u-fukui.ac.jp (M.M.); usyosi@gmail.com (S.Y.); junya-mori@doc.city.sapporo.jp (J.M.); tsawada@belle.shiga-med.ac.jp (T.S.); hmatsu@clin.medic.mie-u.ac.jp (H.M.); kusu@med.kobe-u.ac.jp (S.K.); tomoyam618@gmail.com (T.M.); akikato@med.nagoya-cu.ac.jp (A.K.); h.tabuchi@tsukazaki-eye.net (H.T.); d.nagasato@tsukazaki-eye.net (D.N.); uedatetuo@hotmail.com (T.U.); masahiko@v101.vaio.ne.jp (M.S.); takaoh@shinshu-u.ac.jp (T.H.); t2jujo@marianna-u.ac.jp (T.J.); ymitaymitaymita@yahoo.co.jp (Y.M.); nishigaki.masashi.860@mail.aichi-med-u.ac.jp (M.N.); fwgi0461@ndmc.ac.jp (K.H.); marii.kt@gmail.com (M.S.); 2 Department of Ophthalmology, Faculty of Medical Sciences, University of Fukui, Yoshida 910-1193, Japan; inatani@u-fukui.ac.jp; 3 Department of Ophthalmology, Kurume University School of Medicine, Kurume 830-0011, Japan; 4 Department of Ophthalmology, Sapporo City General Hospital, Sapporo 060-8604, Japan; 5 Department of Ophthalmology, Shiga University of Medical Science, Otsu 520-2192, Japan; 6 Department of Ophthalmology, Mie University Graduate School of Medicine, Tsu 514-8507, Japan; 7 Division of Ophthalmology, Department of Surgery, Kobe University Graduate School of Medicine, Kobe 650-0017, Japan; 8 Department of Ophthalmology, Faculty of Medicine, University of Tsukuba, Tsukuba 305-8576, Japan; 9 Department of Ophthalmology and Visual Science, Nagoya City University Graduate School of Medical Sciences, Nagoya 467-8602, Japan; 10 Department of Ophthalmology, Saneikai Tsukazaki Hospital, Himeji 671-1227, Japan; 11 Department of Ophthalmology, Nara Medical University, Kashihara 634-8522, Japan; 12 Department of Ophthalmology, Tokyo Medical University Hachioji Medical Center, Tokyo 193-0998, Japan; 13 Department of Ophthalmology, Shinshu University School of Medicine, Matsumoto 390-8621, Japan; 14 Department of Ophthalmology, St. Marianna University School of Medicine, Kawasaki 216-8511, Japan; 15 Department of Ophthalmology, Institute of Biomedical Sciences, Tokushima University Graduate School, Tokushima 770-8503, Japan; 16 Department of Ophthalmology, Aichi Medical University, Nagakute 480-1195, Japan; 17 Department of Ophthalmology, National Defense Medical College, Tokorozawa 359-8513, Japan; 18 Department of Ophthalmology, Tachikawa Hospital, Tachikawa 190-8531, Japan; 19 Department of Ophthalmology, Keio University School of Medicine, Tokyo 160-0016, Japan

**Keywords:** aflibercept, prefilled syringe, endophthalmitis

## Abstract

**Background:** Bacterial endophthalmitis is a rare but serious complication of intravitreal injections (IVIs). Prefilled syringes have been introduced to reduce contamination risk during drug preparation. However, whether they lower the incidence of bacterial endophthalmitis compared to vials remains unclear. **Methods:** This retrospective cohort study analyzed aflibercept IVIs performed at 17 clinical centers in Japan between 2015 and 2022. Patients aged ≥20 years who received aflibercept IVIs (vial or prefilled syringe) for age-related macular degeneration, diabetic macular edema, retinal vein occlusion, or myopic choroidal neovascularization were included. Bacterial endophthalmitis was diagnosed based on clinical signs (e.g., rapid vision loss, pain, hypopyon, vitreous opacity). Incidence rates were compared using Fisher’s exact test. **Results:** Among 152,039 injections (43,684 prefilled syringes; 108,355 vials), 12 cases of bacterial endophthalmitis were identified (0.0046% vs. 0.0092%, *p* = 0.53). Poor visual outcomes were associated with *Enterococcus faecalis*, *Streptococcus* spp., and diabetes. **Conclusions:** Although incidence was lower in the prefilled syringe group, the difference was not statistically significant. Detecting a significant difference requires a larger sample. Further studies are needed to confirm the potential benefits of prefilled syringes in reducing endophthalmitis risk.

## 1. Introduction

Intravitreal injection (IVI) of an anti-vascular endothelial growth factor (VEGF) agent is the standard treatment for retinal diseases such as age-related macular degeneration (AMD), diabetic macular edema (DME), retinal vein occlusion (RVO), and myopic choroidal neovascularization (mCNV). With their increasing use worldwide, these injections are also used to treat neovascular glaucoma and retinopathy of prematurity [1]. Moreover, their therapeutic efficacy and safety have been demonstrated in numerous large-scale studies [2,3,4,5,6,7,8], establishing them as the first-choice treatment for retinal diseases. Bacterial endophthalmitis is a major complication of IVI that occurs when bacteria are transmitted to the eye during the injection. Its frequency is reported to be approximately 0.01–0.26%, making it a rare condition [9]. However, once it develops and when the treatment is delayed, it can cause severe visual impairment. Cleanliness is important for preventing endophthalmitis. Iodine-based antiseptics used to disinfect the surgical field reportedly decrease the risk of endophthalmitis and are recommended in the U.S. guidelines for IVIs [10,11]. In addition, wearing a mask during the injection procedure has been reported to decrease this incidence [12].

The formulation of the drug also influences the incidence of bacterial endophthalmitis. The history of the representative anti-VEGF agents used for IVI and the development of their formulations was as follows. Bevacizumab was initially used off-label, primarily as an anti-VEGF drug for IVIs. It was originally a formulation used in cancer chemotherapy. In the United States, large-volume bevacizumab formulations were dispensed aseptically in compounding pharmacies [13]. Previously, an outbreak of post-injection intraocular inflammation due to contamination during dispensing was reported [14,15]. Conversely, ranibizumab was the first drug developed specifically for IVI as a vial formulation. Subsequently, the prefilled syringe formulation was introduced, which is convenient because it avoids the process of manual filling, simplifying the injection procedure. This simplified procedure further contributes to the maintenance of aseptic conditions. Vials require the transfer of a drug solution into a syringe, which can lead to bacterial contamination of the injectable drug. The incidence of culture-positive endophthalmitis reportedly decreased when ranibizumab vial formulation was replaced with a prefilled syringe formulation, suggesting that the use of a prefilled syringe may reduce the risk of contamination [16].

After ranibizumab, aflibercept was the second drug to be administered via IVI. Aflibercept was initially available as a vial formulation; however, it was associated with a higher incidence of endophthalmitis than that with a prefilled syringe formulation of ranibizumab [17]. Subsequently, a prefilled syringe formulation was launched and made available in Japan in June 2020. With the introduction of this new formulation, the incidence of aflibercept-induced endophthalmitis is expected to decrease. A study conducted by Bayer, the manufacturer of aflibercept, based on its own database, found that the frequency of endophthalmitis was lower with the prefilled syringe formulation than with the vial formulation [18]. However, since this database study classified not only bacterial endophthalmitis but also sterile endophthalmitis and mild iritis under the same category, the precise risk of bacterial endophthalmitis remains unclear. We hypothesized that the incidence of bacterial endophthalmitis would be lower when aflibercept is administered via prefilled syringes compared to vial formulations, due to the reduced risk of contamination during the preparation process. To test this hypothesis, we conducted a retrospective cohort study involving multiple centers in Japan, focusing solely on cases of clinically diagnosed bacterial endophthalmitis. The primary objective of this study was to compare the incidence of bacterial endophthalmitis between the prefilled syringe and vial formulations of aflibercept. The secondary objective was to examine the clinical outcomes and causative organisms of endophthalmitis cases that occurred following aflibercept injections.

## 2. Materials and Methods

We conducted a retrospective cohort study across 17 clinical centers in Japan. The University of Fukui Institutional Review Board and ethics committees of other participating facilities (Kurume University, Sapporo City General Hospital, Shiga University, Mie University, Kobe University, University of Tsukuba, Nagoya City University, Saneikai Tsukazaki Hospital, Nara Medical University, Tokyo Medical University Hachioji Medical Center, Shinshu University, St. Marianna University, Tokushima University, Aichi Medical University, National Defense Medical College, and Tachikawa Hospital) approved the study protocol. All the study procedures adhered to the tenets of the Declaration of Helsinki. An opt-out method was adopted at each participating facility to inform patients regarding the study. Each institution posted a notice about the study on its website or bulletin board, allowing patients to decline participation if they wished.

### 2.1. Study Population

We retrospectively reviewed medical records and aggregated the number of intravitreal injections (IVIs) administered between 1 January 2015, and 30 September 2022, categorized by formulation.

Inclusion Criteria:

- Age: ≥20 years;
- Sex: No restrictions;
- Diagnosis: AMD, DME, RVO, or mCNV;
- Administered Drug: Aflibercept (Eylea; Bayer Yakuhin Ltd., Tokyo, Japan), either in vial or prefilled syringe formulation.

Exclusion Criteria:

- Patients who declined participation through the opt-out process.

Among patients who met these criteria, those who developed bacterial endophthalmitis following aflibercept injection were further analyzed for additional parameters such as disease, bacterial culture results, visual acuity changes (at injection, at presentation, and at three months after presentation), and the number of prior injections.

### 2.2. Diagnosis of Bacterial Endophthalmitis

Bacterial endophthalmitis cases were identified based on clinical presentation, regardless of bacterial culture results. Typical clinical signs of bacterial endophthalmitis included rapid-onset vision loss within a few days after injection, ocular pain, ciliary injection, hypopyon, vitreous opacity, retinal vascular occlusion, and extensive retinal hemorrhage. Cases with these findings were classified as bacterial endophthalmitis even if bacterial culture results were negative. Conversely, cases in which differentiation between bacterial and sterile endophthalmitis was uncertain were excluded.

### 2.3. Data Collection

Authors at each site reviewed medical records and collected data on 1 October 2022. All authors had access to information that could identify individual participants during and after data collection. The data collected in this study partially overlap with those from our previous report [17]. Since this study aimed to evaluate the incidence of endophthalmitis per injection, detailed patient data were not collected for cases without endophthalmitis. However, for cases in which bacterial endophthalmitis occurred, additional patient-level information was obtained.

### 2.4. Injection Procedure

IVIs were administered in accordance with the Japanese vitreous injection guidelines at all participating facilities [19]. Oxybuprocaine hydrochloride (0.4% benoxyl ophthalmic solution; Santen Co., Ltd., Osaka, Japan) was used as the anesthetic, and an eyelid speculum was used to stabilize the eyelids. The conjunctival sacs were disinfected using iodine-based disinfectants, and 2 mg/0.05 mL of aflibercept was injected intravitreally.

### 2.5. Statistical Analysis

Statistical analyses were performed using JMP 18.1 software (SAS Institute Inc., Tokyo, Japan). Incidence rates and associated 95% confidence intervals (CIs) were calculated on a per-injection basis. Fisher’s exact test was used to assess the differences between groups. The level of statistical significance was set at *p* < 0.05.

## 3. Results

Table 1 presents the number of aflibercept injections and the incidence of endophthalmitis according to the dosage form. A total of 152,039 eyes were administered aflibercept, of which 108,355 were vial formulations and 43,684 were prefilled syringe formulations. There were 10 cases of endophthalmitis in the vial formulation group and two in the prefilled syringe formulation group. The incidence of endophthalmitis was 0.0092% (1 in 10,835 injections) for vial formulation and 0.0046% (1 in 21,842 injections) for prefilled syringe formulation. The odds ratio showed a trend toward a lower incidence of endophthalmitis with prefilled syringe formulation (odds ratio, 0.49; 95% CI: 0.11–2.26); however, the difference was not statistically significant (*p* = 0.53).

Table 2 details the 12 cases of endophthalmitis; 10 were from the vial formulation group and two were from the prefilled syringe formulation group. Seven vial cases and one prefilled syringe case were culture-positive. *Staphylococcus spp.* was detected in most cases. Among the cases in which vials were used, one each of *α Streptococcus* and *Enterococcus faecalis* were detected. Drug-resistant bacteria were found in patients receiving both vial and prefilled syringe formulations. In terms of visual acuity, six cases returned to pre-injection visual acuity levels; however, six cases remained with marked visual loss.

## 4. Discussion

This is the first study to compare the incidence of endophthalmitis between the vial and prefilled syringe formulations of aflibercept in Japan. Although no statistically significant difference was observed, the incidence of endophthalmitis for prefilled syringe formulations (0.0046%) was approximately half of that for vial formulations (0.0092%). This suggests a potential trend toward a lower incidence with prefilled syringes; however, due to the low overall incidence of endophthalmitis, a definitive conclusion cannot be drawn. Moreover, not all previous reports have demonstrated significantly lower rates of endophthalmitis with prefilled syringe formulations [16,20,21,22]. In a study by Storey et al., which included a large number of cases, no significant difference was observed in the incidence of endophthalmitis. This aligns with our findings, highlighting the challenge of detecting statistically significant differences given the rarity of endophthalmitis.

Recent reports indicate a lower incidence of endophthalmitis than in the past, with a large study published around 2015 reporting an incidence of approximately 1 case per 2000 persons [23,24]. However, reports from 2020 onward indicate an incidence of approximately 1 case per 3000 persons and 1 case per 4000 persons [25,26]. In addition, reports from Japan indicated an incidence rate of less than 1 in 10,000 persons [27,28]. While this declining trend is encouraging, it complicates the ability to assess the impact of specific interventions on endophthalmitis incidence.

Previous studies have collected cases from multiple countries; however, due to the abovementioned regional differences in endophthalmitis incidence, we restricted our data collection to Japan in this study [16]. Furthermore, a longer study period was considered. Although the number of aflibercept injections has increased over the years, other anti-VEGF agents have been successively introduced, making it difficult to predict the future volume of aflibercept use. Hence, we considered the selected study period to be appropriate. Additionally, while a larger dataset could have been obtained using insurance claim records [20], such an approach may have led to misclassification, as aseptic and fungal endophthalmitis might be recorded under the general category of endophthalmitis. To address this, we collaborated with J-CREST, one of the largest retinal clinical research groups in Japan, to recruit multiple participating institutions. The data were collected by researchers at each facility through medical record review, ensuring that conditions misclassified as endophthalmitis were excluded. As a result, our dataset provides a precise representation of bacterial endophthalmitis cases, offering valuable insights into its actual incidence.

One important consideration is the statistical power of our study. Although a large number of injections were analyzed, the extremely low incidence of endophthalmitis inherently limits the ability to detect statistically significant differences. A power analysis indicated that detecting such a difference at a conventional α level of 0.05 with 80% power would require approximately 500,000 injections per group (1,000,000 in total). Although achievable in large-scale databases or national registries, such a sample size is not typically feasible in investigator-initiated clinical studies, highlighting the inherent challenge of evaluating rare adverse events using traditional statistical approaches. Therefore, while our findings suggest a possible difference in endophthalmitis rates, the study was not powered to demonstrate statistical significance.

Several potential confounding factors should be considered when interpreting our findings. One important factor is the underlying retinal disease that required intravitreal injection. Previous studies have reported that the incidence of endophthalmitis may vary depending on the primary condition being treated. For instance, AMD and DME have been associated with a higher risk of endophthalmitis compared to other retinal diseases [29]. As our study did not stratify patients based on the underlying disease, any imbalance in the proportion of DME cases between the two groups could have influenced the observed incidence rates.

Differences in aseptic technique across institutions may also have introduced confounding. As this was a multicenter study, variability likely existed in clinical practices such as the use of perioperative antimicrobials, disinfection protocols, mask use by injecting physicians, and overall cleanliness procedures. For instance, the use of face masks during IVIs has been shown to reduce the risk of endophthalmitis [12], and inconsistencies in this practice could have affected infection rates. However, previous studies suggest that antimicrobial prophylaxis has limited influence on endophthalmitis incidence [9,17,30,31], and all participating centers were expected to follow standardized safety guidelines for intravitreal injections. Although we could not assess the specific procedural details at each site due to limitations in our dataset, the influence of inter-institutional variability is likely limited.

Furthermore, the total number of injections received by each patient may represent another potential confounder. Previous studies have suggested that patients undergoing fewer than three injections are at greater risk of developing endophthalmitis [32]. If the distribution of injection frequency differed between the prefilled syringe and vial groups, this could have biased the observed incidence rates. Our study did not stratify cases based on cumulative injection exposure, which represents a limitation in evaluating this variable. Taken together, while several potential sources of confounding exist, their individual impact is difficult to quantify, and the observed differences should therefore be interpreted with appropriate caution.

In this study, 12 cases of bacterial endophthalmitis were observed. Visual function was preserved in six cases (patient no. 1, 3, 4, 5, 11, 12), whereas the remaining six cases (patient no. 2, 6, 7, 8, 9, 10) experienced severe visual deterioration. Among these, *E. faecalis* and *Streptococcus* spp. were detected in two cases (patient no. 7, 10), both of whom had a poor visual prognosis. These bacteria are known to produce proteases that cause severe retinal damage, resulting in significant visual loss [33,34,35]. Furthermore, two patients with poor visual outcomes (patient no. 8, 9) had diabetes, which has been reported as a risk factor for worse prognosis in endophthalmitis [36]. The association between diabetes and visual prognosis in endophthalmitis warrants further investigation.

It is also important to note that four cases in our study were culture-negative. However, based on clinical presentation, these cases were considered to be bacterial rather than sterile endophthalmitis. Culture positivity rates in bacterial endophthalmitis have been shown to range from 30% to 60% [37], suggesting that negative cultures do not necessarily rule out bacterial infection. A variety of factors, including prior antibiotic use, inadequate sample volume, or fastidious bacterial growth requirements, may contribute to false-negative cultures. Therefore, while microbiological confirmation is valuable, clinical diagnosis remains crucial in managing bacterial endophthalmitis.

*E. faecalis* and *Streptococcus* spp. are rarely detected in the conjunctival sac and are not typical skin or conjunctival commensals. Previous studies have reported that Enterococcus spp. may persist in the conjunctival sac in some patients [38]; however, such cases are uncommon. Given their low prevalence on the ocular surface, the presence of these bacteria in the vitreous cavity suggests the possibility of contamination during the injection process. In contrast, *Staphylococcus* spp., a common skin and conjunctival bacterium, is frequently identified as a causative agent of post-injection endophthalmitis [38,39].

If contamination occurs during the transfer of drugs from a vial to a syringe, the main contaminants are expected to be skin and oral commensal bacteria. Vial lids may become contaminated through hand contact or droplets expelled during conversation, and bacteria entering the drug solution via a contaminated vial lid can subsequently enter the eye during injection. Even if the ocular surface is sterilized with iodine disinfection, contamination introduced in this manner cannot be prevented. Once the vitreous cavity is contaminated, antimicrobial agents may not reach effective concentrations, and post-injection topical antibiotics have limited efficacy [40]. Therefore, measures should be taken to minimize vial contamination, such as cleaning the vial lid with alcohol before drug transfer.

Notably, among patients who received prefilled syringe formulations, no cases of bacterial endophthalmitis were associated with bacterial species typically introduced during the injection process.

To summarize, this study did not find a statistically significant difference in the incidence of endophthalmitis between prefilled syringes and vial formulations of aflibercept. Therefore, these results alone do not provide sufficient evidence to conclude that prefilled syringes are associated with a lower incidence of endophthalmitis. Given the extremely low incidence of endophthalmitis, detecting a statistically significant difference would require an estimated sample size of approximately 500,000 injections per group (1,000,000 in total) to achieve 80% power at a conventional α level of 0.05. Therefore, while this study suggests a potential difference in endophthalmitis incidence between formulations, a definitive conclusion could not be drawn.

Bacterial endophthalmitis remains a serious complication of intravitreal injections, with significant variability in visual outcomes. In this study, visual function was preserved in six cases, while six cases experienced severe visual deterioration. Poor outcomes were observed in cases involving *E. faecalis* or *Streptococcus* spp., as well as in diabetic patients, consistent with previous reports. This highlights the need for early recognition and aggressive treatment in high-risk cases.

Avoiding endophthalmitis is important when administering IVIs. Various anti-VEGF agents are currently available, and ophthalmologists must select the most appropriate agent. It is important to select a drug that will most likely improve vision. If more than one drug is expected to have an equivalent therapeutic effect, a prefilled syringe formulation should be considered as a means of minimizing procedural contamination risks. However, some of the newly introduced anti-VEGF agents such as faricimab are currently available only in vial form. When using these agents, it is necessary to pay even greater attention to aseptic techniques to prevent endophthalmitis.

Future research should focus on larger-scale registry-based studies or meta-analyses to further clarify differences in endophthalmitis incidence between different injection techniques and formulations. Additionally, controlling for potential confounding factors, such as cumulative injection exposure, would allow for a more precise evaluation of the risks associated with different drug delivery methods.

## Figures and Tables

**Table 1:** Number of injections and the incidence of endophthalmitis in the two groups according to aflibercept formulation.

Vial		Prefilled Syringe		Odds Ratio	
Injections	Cases (Incidence)	Injections	Cases (Incidence)	(95% Confidence Interval)	*p* Value
108,355	10 (0.0092%)	43,684	2 (0.0046%)	0.49 (0.11–2.26)	0.53
	1 in 10,835 injections		1 in 21,842 injections		

**Table 2:** Characteristics of the 12 patients who experienced endophthalmitis.

					Visual Acuity (log MAR)			
Patient No	Dosage Form	Disease	Culture Results	Resistance to Used Antibiotics	At Injection	At Presentation	At 3 Months After Presentation	No. of Injections
1	vial	AMD	*Staphylococcus capitis*	Not tested	0.70	CF	0.70	5
2	vial	AMD	*Staphylococcus aureus*	Yes	0.22	CF	0.82	13
3	vial	AMD	negative	-	0.30	HM	0.40	5
4	vial	AMD	*Staphylococcus capitis*	Not tested	0.70	CF	0.70	5
5	vial	AMD	negative	-	0.00	1.70	0.05	18
6	vial	AMD	*Staphylococcus epidermidis*	Yes	0.00	HM	0.40	6
7	vial	RVO	*αsteptococcus*	No	0.00	LP	LP	13
8	vial	DME	*Staphylococcus epidermidis*	Yes	0.52	CF	1.10	9
9	vial	DME	negative	-	0.30	CF	0.70	5
10	vial	mCNV	*Enterococcus faecalis*	No	0.82	LP	HM	2
11	prefilled syringe	AMD	*Staphylococcus epidermidis*	Yes	0.00	HM	0.00	2
12	prefilled syringe	RVO	negative	-	0.70	1.20	0.80	32

HM, hand motion; CF, counting finger; LP, light perception.

## Data Availability

The datasets generated and/or analyzed during the current study are available from the corresponding author upon reasonable request.
